# Glycemic and lipid variability for predicting complications and mortality in diabetes mellitus using machine learning

**DOI:** 10.1186/s12902-021-00751-4

**Published:** 2021-05-04

**Authors:** Sharen Lee, Jiandong Zhou, Wing Tak Wong, Tong Liu, William K. K. Wu, Ian Chi Kei Wong, Qingpeng Zhang, Gary Tse

**Affiliations:** 1 Cardiovascular Analytics Group, Laboratory of Cardiovascular Physiology, Hong Kong, China; 2 School of Data Science, City University of Hong Kong, Hong Kong, China; 3 School of Life Sciences, Chinese University of Hong Kong, Hong Kong, China; 4 Tianjin Key Laboratory of Ionic-Molecular Function of Cardiovascular disease, Department of Cardiology, Tianjin Institute of Cardiology, Second Hospital of Tianjin Medical University, Tianjin, 300211 China; 5 Li Ka Shing Institute of Health Sciences, The Chinese University of Hong Kong, Hong Kong, China; 6 Department of Pharmacology and Pharmacy, University of Hong Kong, Pokfulam, Hong Kong, China; 7 Medicines Optimisation Research and Education (CMORE), UCL School of Pharmacy, London, UK; 8 Faculty of Health and Medical Sciences, University of Surrey, Guildford, GU2 7AL UK

## Abstract

**Introduction:**

Recent studies have reported that HbA1c and lipid variability is useful for risk stratification in diabetes mellitus. The present study evaluated the predictive value of the baseline, subsequent mean of at least three measurements and variability of HbA1c and lipids for adverse outcomes.

**Methods:**

This retrospective cohort study consists of type 1 and type 2 diabetic patients who were prescribed insulin at outpatient clinics of Hong Kong public hospitals, from 1st January to 31st December 2009. Standard deviation (SD) and coefficient of variation were used to measure the variability of HbA1c, total cholesterol, low-density lipoprotein cholesterol (LDL-C), high-density lipoprotein cholesterol (HDL-C) and triglyceride. The primary outcome is all-cause mortality. Secondary outcomes were diabetes-related complications.

**Result:**

The study consists of 25,186 patients (mean age = 63.0, interquartile range [IQR] of age = 15.1 years, male = 50%). HbA1c and lipid value and variability were significant predictors of all-cause mortality. Higher HbA1c and lipid variability measures were associated with increased risks of neurological, ophthalmological and renal complications, as well as incident dementia, osteoporosis, peripheral vascular disease, ischemic heart disease, atrial fibrillation and heart failure (*p* <  0.05). Significant association was found between hypoglycemic frequency (*p* <  0.0001), HbA1c (*p* <  0.0001) and lipid variability against baseline neutrophil-lymphocyte ratio (NLR).

**Conclusion:**

Raised variability in HbA1c and lipid parameters are associated with an elevated risk in both diabetic complications and all-cause mortality. The association between hypoglycemic frequency, baseline NLR, and both HbA1c and lipid variability implicate a role for inflammation in mediating adverse outcomes in diabetes, but this should be explored further in future studies.

**Supplementary Information:**

The online version contains supplementary material available at 10.1186/s12902-021-00751-4.

## Introduction

There is an increasing global prevalence of diabetes mellitus, with over 400 million people around the world currently suffering from the disease [1]. Diabetes mellitus can lead to a variety of complications affecting the cardiovascular, neurological, renal and other systems, placing significant burdens on healthcare systems globally [2–4]. Given the aging population, an increasing proportion of diabetic patients are elderly with multiple comorbidities, leading to a call for a more personalized and patient-centered approach in diabetic management over recent years [5–7]. This raises the need for new parameters for monitoring diabetes, other than blood glucose, to improve the sensitivity towards the disease progression across different organ systems [8–12]. Diabetic patients who are on insulin are more advanced in the disease life course, and as such are at a higher risk of complications and death. Recently, HbA1c and lipid variability have attracted attention in its potential use for diabetic monitoring and risk stratification for adverse outcomes. However, existing studies focused on cardiovascular events and mortality [13–15]. Although the exact pathways of pathogenesis by HbA1c and different lipid variability are unclear and appear to be divergent, the resulting chronic inflammation and endothelial dysfunction may have led to the presentation of systemic complications in diabetes [16–18]. Other suggest that raised variability in biomarkers reflects lifestyle changes, incomplete treatment adherence, pharmacotherapy prescribed, and generalized frailty [19–21]. Random survival forest (RSF) is a class of machine learning algorithms for survival analysis [22]. The advantage of RSF is that it can reduce the variance and bias within the input variables and automatically consider nonlinear effects and high-level interactions among these variables. Thus, RSF can be applied to select and rank variables based on their importance. In this study, we aim to evaluate the predictive value of glycemic and lipid variability towards a wide range of adverse outcomes in diabetes and that risk prediction is more accurate using RSF.

## Methods

### Study population

The present study is a territory-wide observational study that collects data from 43 public hospitals in Hong Kong. The study was approved by The Joint Chinese University of Hong Kong – New Territories East Cluster Clinical Research Ethics Committee. It was performed in accordance with the Declaration of Helsinki as well as relevant guidelines and regulations. The cohort consists of diabetic patients who have been prescribed insulin from outpatient clinics of any public hospitals managed by the Hong Kong Hospital Authority between January 1st to December 31st, 2009. Patients were not required to be on insulin for a minimum period. Through the Clinical Data Analysis and Reporting System (CDARS), a healthcare database that integrates patient information across all publicly-funded hospitals and their associated ambulatory and primary care clinics in Hong Kong to establish holistic medical records, the cohort was identified, and the data was extracted. The system has been utilized for epidemiological research by multiple research teams, including our team, in the past [23–26].

### Patient data

Clinical outcomes, patient characteristics and pharmacological treatment details were extracted. The patient outcomes from January 1st, 2009 to December 31st, 2019 were extracted. Patients were followed up from January 1st, 2009 to either death, or December 31st, 2019. The primary outcome is all-cause mortality, and the secondary outcomes, as defined by their International Classification of Disease, Ninth Edition (ICD-9) codes (Supplementary Table 1), include: 1) neurological, ophthalmological and renal diabetic complications, 2) dementia, 3) osteoporosis, 4) peripheral vascular disease (PVD), 5) intracranial hemorrhage, 6) ischemic stroke and transient ischemic attack (TIA), 7) ischemic heart disease (IHD), acute myocardial infarction (AMI) and heart failure (HF), 8) atrial fibrillation (AF).

The extracted parameters of patient details were summarized in Supplementary Table 2. The duration of diabetes at baseline was extracted based on the following three criteria, selected based on whichever is earlier: 1) earliest ICD-9 coding of diabetes mellitus; 2) earliest HbA1c > 6.5 mmol/L; 3) earliest fasting blood glucose > 7 mmol/L. The mean daily dose of anti-diabetic and cardiovascular medications drug classes was reported. The mean daily dose is derived from multiplying the daily dose frequency against the drug dose, then averaged by all patients that were prescribed drugs of the specific drug class. In terms of biochemical data, baseline neutrophil-lymphocyte ratio (NLR) was derived from dividing the baseline absolute neutrophil count by the lymphocyte count. To assess glycemic and lipid variability, data for the following variables between January 1st, 2004 and December 31st, 2008 were obtained: 1) HbA1c, 2) total cholesterol, 3) high-density lipoprotein cholesterol (HDL-C), 4) low-density lipoprotein cholesterol (LDL-C), 5) total triglyceride. LDL-C includes both findings from direct and calculated measurements. Furthermore, the frequency of hypoglycemic episodes across the entire follow-up period from laboratory tests taken during outpatient, inpatient and accident and emergency settings was extracted. Each episode is defined by random or fasting blood glucose < 3.9 mg/mmol. Additionally, the presence of anemia, defined by hemoglobin < 13 g/dL and < 12 g/dL for male and female patients respectively, were extracted. The presence of iron deficiency, defined by ferritin < 67.4 pmol/L, was also extracted. Only patients with three or more measurements for the specific parameter were included for the variability analysis of the respective parameter.

### Statistical analysis

Temporal variability was examined using the derivation of standard deviation (SD) and coefficient of variation (CV). CV was given by the temporal SD divided by the temporal mean, then multiplied by 100. Univariate Cox regression was applied to identify significant predictors from demographic variables, biochemical parameters, and anti-diabetic agents prescribed for the various adverse outcomes. GLP agonists and meglitinide were excluded from the analysis due to the limited number of patients prescribed with the drugs. The hazard ratio (HR) and 95% confidence interval (CI) were presented for each predictor. Patients with missing data were excluded from the analysis for that particular variable. Predictors with *P*-value < 0.10 under univariate analysis for all-cause mortality is then selected to undergo multivariate Cox regression. Patients were excluded from the multivariate analysis if they do not have at least three measurements for the assessment of variability, or if there are missing data in any of the significant predictors found under univariate cox analysis.

To examine the inter-relationship between HbA1c variability, intermittent hypoglycemia, and chronic inflammation, Gaussian, and Poisson regression were used to assess the correlations of HbA1c variability against baseline NLR and hypoglycemia frequency respectively. Gaussian regression was also used to assess the association between the lipid parameters, and lipid indices against baseline NLR. Gaussian regression is a non-parametric method to assess the association between two continuous variables, hence suitable to assess the inter-relationship between HbA1c/ lipid variability and baseline NLR. Poisson regression is a model that allows the assessment between a count variable, in this case hypoglycemic frequency, and continuous variables. Odds ratio (OR) is reported for both Poisson and Gaussian regression. Statistical significance is defined as *P*-value < 0.05. Statistical analyses were performed using RStudio software (Version: 1.1.456) and Python (Version: 3.6).

### Development of a regularized and weighted random survival forests model

Random survival forests (RSF) [22] is machine-learning modelling technique that can capture complex survival data structures and overcome the restrictive assumption of Cox proportional model to better uncover the nonlinear relationships between covariates and the time of event outcome. In contrast, assumptions about specialized basis functions in Cox models are not efficient for assessing the nonlinear effects by transformations or expanding the design matrix. The RSF model is constructed with an ensemble tree method for the analysis of right-censored survival data, extended from Breiman’s random forests. It is an efficient ensemble learning method by injecting randomization into base learning processes and has become one of the most efficient models in survival analysis.

**Fig. 1:**
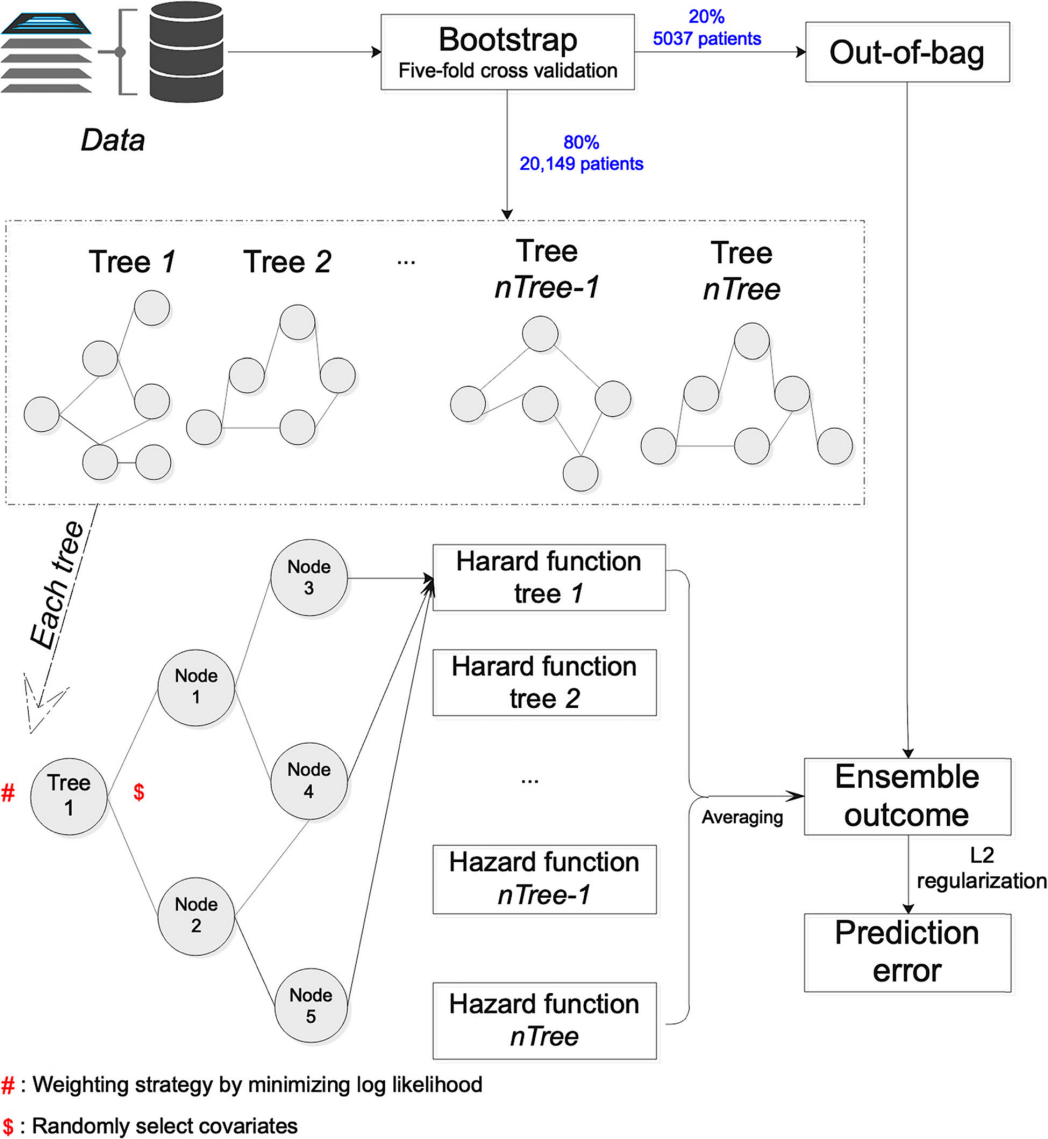
Workflow of regularized and weighted random survival forests model

In this study, the time for RSF survival learning is defined as the duration from baseline date to event presentation or mortality/study end date if no event presentation before mortality and study end. More specifically, as shown in Fig. 1 for the workflow of regularized and weighted random survival forests model that we developed to predict mortality and complication outcomes, the regularized and weighted RSF model can estimate the forest hazard survival function with an averaging procedure through tree ensembling approach. The ensembling procedure assigns equal weights on different survival decision trees. In this study, we consider the heterogeneity among the multiple ensembled survival decision trees to give their predictions [27] and propose to fill this gap by adopting a weighted averaging strategy as shown in Fig. 1 to assign different weights to different survival trees. The assigned weights for different survival trees were learned with the objective of minimizing the overall loss function (e.g., log likelihood we used in this study). To avoid the problem of overfitting, we adopted a L2 regularization strategy and the optimal regularization strength parameter for the log likelihood loss function in the model. The regularization parameters were determined by five-fold cross validation on the training set (80% patients in the cohort). Different values for the weighting and regularization parameters were tested, and we selected those with the best results. In this way, we obtain a regularized and weighted RSFs which consider heterogeneity among those survival decision trees by weighting strategy and avoid overfitting by adding L2 regularization to predict the outcomes of mortality and different complications.

In addition, with the developed RSF machine learning model, we can provide interpretations about the learning results by estimating the relative importance and minimal depth approaches in the learned survival trees for predicting the mortality and complication outcomes. A variable importance approach was adopted based on standard bootstrap theory to investigate the predictive strength of the associated risk factors. The importance value for the variable of interest is the prediction error for the original ensemble event-specific cumulative probability function (obtained when each out-of-bag instance is just dropped down its in-bag competing risks tree) subtracted from the prediction error for the new ensemble obtained using randomizing assignments of the variable [28, 29]. The prediction errors are computed using squared loss. Larger importance value indicates higher predictive strength of the variable, whereas zero or negative values identify nonpredictive variables. Minimal depth approach [30] is an alternative method to measure the predictive strength of variables in random survival forests model, which ranks variables through the inspection of the forest construction process since in tree structured models’ variables with high impact on the prediction are those that most frequently split nodes nearest to the root node where they partition the largest samples (higher impact). Minimal depth approach identifies important variables by averaging the depth of the first split for each variable over all trees within the final forest to predict the mortality and different complication outcomes.

Significant variables from univariate Cox regression were used as inputs into the regularized and weighted RSF model. The performance of the model is compared with several baseline models, including the RSF and the Cox model. Missing values are “-1” padded. The model is trained on the training set with a five-fold cross validation approach. Model’s discrimination performance is accessed by Harrell’s C-index, which is a generalization of the area under the receiver operating characteristic curve (AUC) that can handle right-censored data to estimate the efficiency of the model at ranking survival times. Comparisons on the performance of the model with several baselines including RSF and Cox regression model were also provided. The codes have been uploaded to Github (https://github.com/jadonzhou/Glycemic-and-lipid-variability-for-DM-prediction.git).

## Results

### Clinical and biochemical characteristics

The study cohort consists of 25,186 patients (mean age = 63.0, interquartile range [IQR] of age = 15.1 years, male = 50.4%, type 1 diabetes mellitus = 7.37%, baseline diabetes duration = 2.84 ± 2.54 years, total duration = 69,332 patient-years, daily insulin dosage: 20.2 ± 12.6 units). A graphical illustration of the methodology is shown in Fig. 1. Tables 1 and 2 displays the discrete and continuous baseline characteristics of the study cohort respectively. The most prevalent pre-existing comorbidity is hypertension (35.6%), followed by ophthalmological conditions (32.2%), and IHD (16.2%). Other baseline details include drug descriptions are shown in the Supplementary Appendix.

**Table 1:** Discrete Baseline Characteristics

	Patient Percentage (%)
Demographic	
Male	50.4
Type 1 Diabetes Mellitus	7.37
Comorbidities	
Hypertension	35.6
Ophthalmological Complications	32.2
Ischemic Heart Disease	16.2
Ischemic Stroke and Transient Ischemic Attack	11.8
Heart Failure	9.8
Chronic Renal Disease	8.8
Chronic Liver Disease	5.8
Acute Myocardial Infarction	5.1
Chronic Obstructive Pulmonary Disease	3.5
Anti-diabetic Medication	
Biguanide	57.6
Sulphonylurea	41.5
Thiazolidinedione	3.5
Alpha-Glucosidase	3.0
Dipeptidyl Peptidase-4 Inhibitor	0.4
Glucagon-Like Peptide-1 Receptor Agonist	< 0.1
Cardiovascular medication	
Angiotensinogen-Converting Enzyme Inhibitor/ Angiotensin-Receptor Blocker	59.8
Calcium Channel Blocker	43.6
Lipid-Lowering Agents	42.4
Aspirin	36.2
Diuretic	29.2
Beta-Adrenergic Receptor Blocker	28.1

**Table 2:** Continuous Baseline Characteristics

	Mean	Standard Deviation
*Urinalysis*		
Albumin/Creatinine Ratio (mg/mmol)	38.1	121
Creatinine Clearance (ml/min)	54.1	35.9
Spot Protein (g/d)	1.17	1.96
Spot Albumin (mg/L)	170	545
Spot Glucose (mmol/L)	12.5	6.68
24-h Total Protein (g/d)	1.17	1.97
24-h Total Albumin (mg/d)	271	695
*Baseline Blood Test*		
Fasting Glucose (mmol/L)	8.96	3.75
Random Glucose (mmol/L)	12.3	7.47
HbA1c (%)	8.56	1.94
Total Cholesterol (mmol/L)	4.74	1.12
High Density Lipoprotein (HDL) Cholesterol (mmol/L)	1.24	0.403
Calculated Low Density Lipoprotein (LDL) Cholesterol (mmol/L)	2.74	0.927
Direct LDL Cholesterol (mmol/L)	2.80	0.925
Triglyceride (mmol/L)	1.80	1.72
*Renal Function Test*		
Creatinine (umol/L)	144	159
Sodium (mmol/L)	139	3.33
Potassium (mmol/L)	4.31	0.506
Urate (umol/L)	0.408	0.129
Urea (mmol/L)	8.82	6.04
*Liver Function Test*		
Albumin (g/L)	39.2	5.56
Alanine Aminotransferase (ALT) (U/L)	24.3	21.6
Alkaline Phosphatase (ALP) (U/L)	85.2	47.0
Total Bilirubin (umol/L)	11.3	8.98
Total Protein (g/L)	74.4	7.13
*Complete Blood Count*		
Hemoglobin (g/dL)	12.5	1.99
Mean Corpuscular Hemoglobin (MCH) (pg)	29.7	2.95
Mean Corpuscular Hemoglobin Concentration (MCHC) (g/dL)	34.0	0.952
Mean Corpuscular Volume (MCV) (fL)	87.2	7.44
Hematocrit (L/L)	0.376	0.539
Basophil (×10^9^/L)	0.029	0.042
Eosinophil (×10^9^/L)	0.223	0.235
Lymphocyte (×10^9^/L)	1.87	0.867
Monocyte (×10^9^/L)	0.538	0.266
Neutrophil (×10^9^/L)	5.47	2.79
Platelet (×10^9^/L)	256	83.3
Red Blood Cell (× 10^12^/L)	4.26	0.740
White Blood Cell (×10^9^/L)	8.09	2.91

### Anti-diabetic drug classes and outcomes

Different classes of anti-diabetic agents are associated with adverse outcomes differently. Thiazolidinedione lowers the risk of neurological complications (HR = 0.718, 95% CI = [0.539, 0.956], *p* = 0.023) and HF (HR = 0.72, 95% CI = [0.54, 0.96], *p* <  0.0001), whilst biguanide only lowers the risk of HF (HR = 0.62, 95% CI = [0.56, 0.68], *p* <  0.0001). The risk for adverse cardiovascular events were raised by sulphonylurea, biguanide, and alpha-glucosidase inhibitor. Sulphonylurea is associated with an increased risk of renal complications (HR = 1.29, 95% CI = [1.22, 1.36], *p* <  0.0001) and dementia (HR = 1.22, 95% CI = [1.08, 1.39], *p* = 0.002), whilst biguanide is related to ophthalmological complications (HR = 1.09, 95% CI = [0.937, 1.26], *p* <  0.0001).

### Adverse outcome and predictors

The characteristics of the adverse outcomes and biochemical predictors are detailed in Tables 3 and 4 respectively. Anemia occurred in 39.1% (*n* = 9848) of the cohort, with iron deficiency presented in 9.76% of the 2100 patients with ferritin measured. Throughout the study period, 12,372 incidences of death took place (male = 52.6%, age of death = 69.7 ± 12.0). The most common adverse outcomes were death (49.1%), renal (21.4%), and ophthalmological diabetic complications (18.7%). Ophthalmological (onset age = 62.8 ± 11.9), neurological (onset age = 64.2 ± 11.9) and renal diabetic complications (onset age = 66.5 ± 12.2) had the earliest onset, whilst osteoporosis (onset age = 72.1 ± 11.3) and dementia (onset age = 74.4 ± 8.30) occurred latest on average, patients in the present cohort experience 1.74 ± 1.72 adverse outcomes.

**Table 3:** Adverse Outcome Characteristics

Outcome	Number of events	Incidence rate	Age of onset	Number of pre-existing comorbidities	Mean onset in follow-up (days)
Mortality	12,372	49.12%	69.7 ± 12.0	2.71 ± 1.66	3056 ± 1396
Renal	5389	21.40%	66.5 ± 12.2	3.51 ± 1.67	3500 ± 1367
Ophthalmological	4705	18.68%	62.8 ± 11.9	3.11 ± 1.81	3590 ± 1296
Ischemic Heart Disease	4532	17.99%	66.8 ± 11.6	3.70 ± 1.74	3700 ± 1119
Acute myocardial infarction	3178	12.62%	68.3 ± 11.1	4.15 ± 1.59	3882 ± 859
Neurological	1861	7.39%	64.2 ± 11.9	4.03 ± 1.77	3952 ± 835
Atrial Fibrillation	1846	7.33%	70.4 ± 10.3	3.75 ± 1.74	3993 ± 715
Heart Failure	1810	7.19%	68.9 ± 11.4	4.61 ± 1.45	3993 ± 711
Ischemic Stroke	1350	5.36%	69.2 ± 10.9	3.55 ± 1.79	4037 ± 634
Intracranial Hemorrhage	1049	4.17%	68.4 ± 11.7	3.45 ± 1.67	4075 ± 530
Dementia	952	3.78%	74.4 ± 8.30	3.37 ± 1.68	4077 ± 535
Peripheral vascular disease	711	2.82%	66.6 ± 12.4	4.39 ± 1.85	4094 ± 505
Osteoporosis	275	1.09%	72.1 ± 11.3	3.01 ± 1.68	4146 ± 299

**Table 4:** Biochemical Predictor Characteristics

Predictors	Mean	Standard Deviation
*HbA1c*		
Baseline (%, *n* = 24,064)	8.56	1.94
Mean (%, *n* = 22,625)	8.64	1.36
Standard Deviation	1.28	0.851
Coefficient of Variation	14.5	8.76
*Total Cholesterol (TC)*		
Baseline (mmol/L, *n* = 23,532)	4.74	1.12
Mean (mmol/L, *n* = 20,445)	4.82	0.871
Standard Deviation	0.663	0.459
Coefficient of Variation	13.5	7.95
*High Density Lipoprotein-Cholesterol (HDL-C)*		
Baseline (mmol/L, *n* = 23,178)	1.24	0.402
Mean (mmol/L, *n* = 19,303)	1.25	0.362
Standard Deviation	0.161	0.100
Coefficient of Variation	1.24	0.403
*Low Density Lipoprotein-Cholesterol (LDL-C)*		
Baseline (mmol/L, *n* = 32,075)	1.24	0.913
Mean (mmol/L, *n* = 18,803)	2.78	0.734
Standard Deviation	0.553	0.359
Coefficient of Variation	20.3	12.5
*Triglyceride*		
Baseline (mmol/L, *n* = 23,518)	1.80	1.72
Mean (mmol/L, *n* = 20,398)	1.86	1.43
Standard Deviation	6.90	1.15
Coefficient of Variation	30.8	17.8
*Other Tests*		
Baseline NLR	3.80	4.16
Baseline Hemoglobin Count (g/dL)	12.5	1.99
Hypoglycemia Frequency	0.537	1.38

The number of patients included for the calculation of mean is the same as the number of patients included for the calculation of standard deviation and coefficient of variation

Multivariate Cox regression analysis was applied to 7913 patients from the study cohort. The multivariate Cox regression for all-cause mortality is presented in Table 5. Mean HbA1c was found to be protective against mortality in univariate analysis (HR = 0.964, *p* <  0.0001), but became predictive on multivariate analysis. However, after adjusting for hematological malignancies, iron deficiency status and lipid-lowering drug use (*n* = 652), HbA1c mean and variability did not remain significant predictors. Amongst the lipid predictors (*n* = 7913), only HDL-C mean (HR = 0.60, 95% CI = [0.51, 0.71], *p* <  0.0001) and SD (HR = 2.18, 95% CI = [1.51, 3.14], *p* <  0.0001) remained significant after adjusting for cancer status and lipid-lowering agent use.

**Table 5:** Multivariate Cox Regression of All-Cause Mortality

Predictor	Hazard Ratio (HR)	95% Confidence Interval (CI)	*P*-Value
Age	1.04	[1.03, 1.04]	**< 0.0001**
Male	1.18	[1.11, 1.27]	**< 0.0001**
Diabetes Duration	0.956	[0.943, 0.970]	**< 0.0001**
*HbA1c*			
Mean	1.09	[1.04, 1.15]	**< 0.001**
Standard Deviation	1.10	[0.825, 1.47]	0.511
Coefficient of Variation	0.998	[0.973, 1.02]	0.869
*Total Cholesterol (TC)*			
Mean	1.14	[0.994, 1.30]	0.061
Standard Deviation	0.787	[0.501, 1.24]	0.299
Coefficient of Variation	1.02	[1.00, 1.05]	**0.050**
*High Density Lipoprotein-Cholesterol (HDL-C)*			
Mean	0.603	[0.513, 0.708]	**< 0.0001**
Standard Deviation	2.19	[1.52, 3.14]	**< 0.0001**
*Low Density Lipoprotein-Cholesterol*			
Mean	0.916	[0.811, 1.03]	0.157
Standard Deviation	1.19	[0.866, 1.64]	0.281
Coefficient of Variation	0.992	[0.983, 1.00]	0.062
*Triglyceride (TG)*			
Baseline	0.996	[0.979, 1.01]	0.694
Mean	1.06	[0.993, 1.14]	0.080
Standard Deviation	0.932	[0.851, 1.02]	0.126
Coefficient of Variation	0.998	[0.995, 1.00]	0.190
*Other Tests*			
Baseline Neutrophil-Lymphocyte Ratio	1.01	[1.01, 1.02]	**< 0.001**
Baseline Hemoglobin Count	0.911	[0.889, 0.934]	**< 0.0001**
Baseline Anemia	1.08	[0.981, 1.19]	0.119
Hypoglycemia Frequency	1.03	[1.01, 1.05]	**0.002**
*Anti-Diabetic Agent*			
Sulphonylurea	1.08	[1.02, 1.16]	**0.015**
Biguanide	0.616	[0.575, 0.660]	**< 0.0001**
Dipeptidyl peptidase-4 Inhibitor	0.706	[0.424, 1.18]	0.181
Thiazolidinedione	0.885	[0.761, 1.03]	0.110

In terms of prediction of secondary outcomes, the predictors were similar to those for all-cause mortality and are summarized in Supplementary Table 3. HbA1c variability is predictive of the adverse outcomes besides osteoporosis, ischemic stroke, and AMI. HbA1c CV is mildly protective of IHD (HR = 0.996, 95% CI = [0.993, 1.00], *p* = 0.046). In terms of lipid predictors, elevated mean total cholesterol is predictive of most adverse outcomes, except for AF (HR = 0.889, 95% CI = [0.838, 0.943], *p* <  0.0001). Increased mean HDL-C lowers the risk for adverse outcomes, except for osteoporosis (HR = 1.78, 95% CI = [1.29, 2.44], *p* <  0.001). Heterogenous predictions were noted for HDL-C variability and mean LDL-C. By contrast, increased LDL-C variability predicts an increased risk for various adverse outcomes. In terms of the predictiveness of triglyceride level, both its value and variability were found to be predictive of different adverse outcomes, except for CV of triglyceride being protective against osteoporosis (HR = 0.990, 95% CI = [0.981, 0.998], *p* = 0.020). Baseline NLR and frequency of hypoglycemic episodes were predictive for a similar set of adverse outcomes, where they increase the risk for PVD), HF, and all-cause mortality, but were associated with a lower risk for ophthalmological complications.

### The relationship between NLR, frequency of hypoglycemic episodes and glycemic variability

The average number of hypoglycemic episodes experienced is 0.54 ± 1.38, and the mean baseline NLR is 3.80 ± 4.16. Baseline mean value of HbA1c was 8.56 ± 1.94%. Variability, represented by SD and CV, are 1.28 ± 0.851 and 14.5 ± 8.76 respectively. HbA1c and lipid variability were significantly associated with baseline NLR with cancer status and aspirin use adjusted, and the associations were summarized in Table 6. Similarly, HbA1c variability was also found to be positively correlated with hypoglycemic frequency (SD: OR = 1.13, 95% CI = [1.12, 1.16], *p* < 0.0001; CV: OR = 1.02, 95% CI = [1.02, 1.02], *p* < 0.0001). Additionally, triglyceride SD is positively correlated with both LDL-C (SD: OR = 1.86, 95% CI = [1.78, 1.93], *p* < 0.0001; CV: OR = 1.02, 95% CI = [1.02, 1.02], *p* < 0.0001) and HDL-C (OR = 2.92, 95% CI = [2.48, 3.43], *p* < 0.0001) variability. After exclusion of calculated LDL-C measurements, the significant association between LDL-C variability and triglyceride SD remains (SD: OR = 1.90, 95% CI = [1.79, 2.02], *p* < 0.0001; CV: OR = 1.02, 95% CI = [1.02, 1.02], *p* < 0.0001).

**Table 6:** Significant associations between HbA1c/ lipid variability with baseline neutrophil-lymphocyte ratio

HbA1c/ Lipid Variability	Hazard ratio [95% Confidence Interval]	*P*-Value
HbA1c: SD	1.01 [1.01, 1.01]	< 0.0001
HbA1c: CV	1.13 [1.10, 1.17]	< 0.0001
HDL-C: SD	1.00 [1.00, 1.00]	< 0.0001
HDL-C: CV	1.19 [1.15, 1.23]	< 0.0001
Triglyceride: CV	1.08 [1.01, 1.16]	0.019
Total Cholesterol: SD	1.01 [1.00, 1.01]	< 0.0001
Total Cholesterol: CV	1.10 [1.07, 1.13]	< 0.0001

The analysis was adjusted to cancer status and aspirin use

*SD* Standard deviation, *CV* Coefficient of variation, *HDL-C* High density lipoprotein-cholesterol

### Survival learning results

**Fig. 2:**
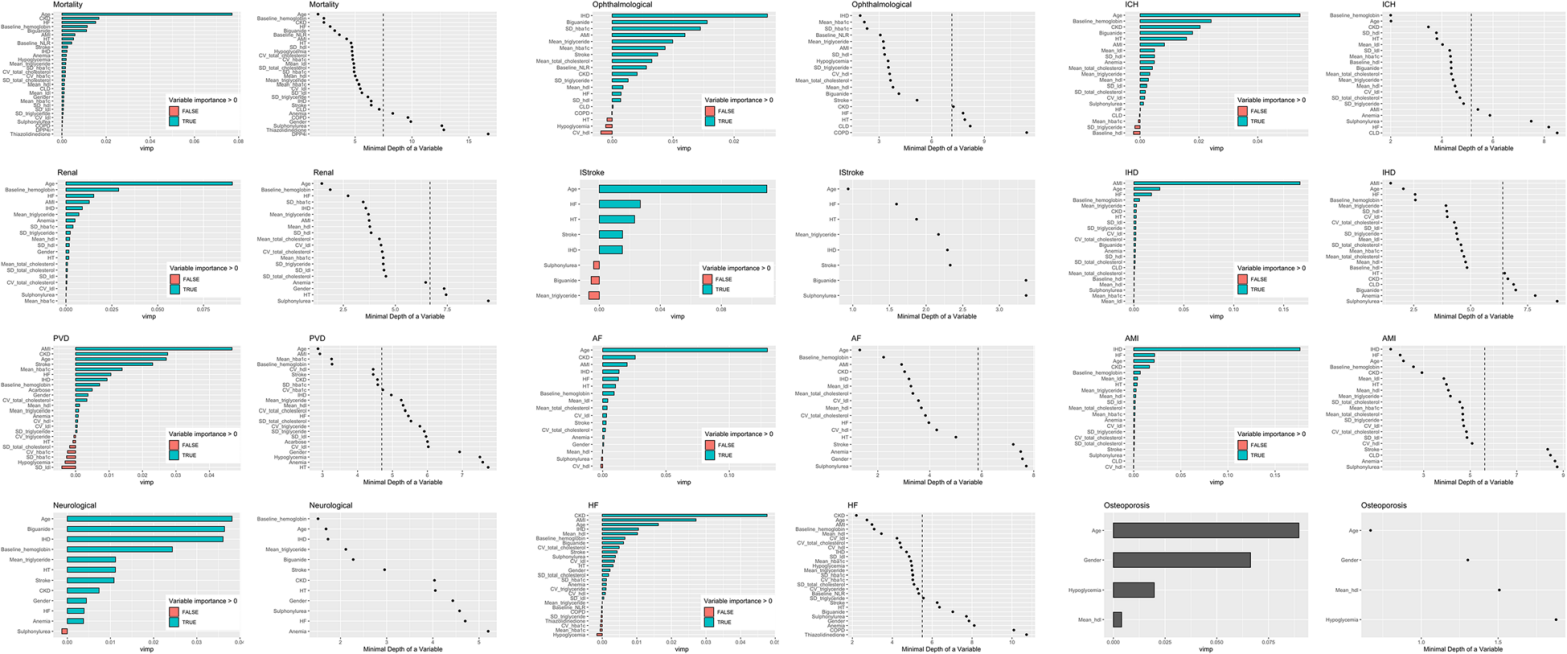
**a** Importance ranking and minimal depth of significant univariable variables to predict mortality, renal, PVD, and neurological complications using regularized and weighted random survival forests model. **b** Importance ranking and minimal depth of significant univariable variables to predict ophthalmological, ischemic stroke, AF, and HF complications using regularized and weighted random survival forests model. **c** Importance ranking and minimal depth of significant univariable variables to predict ICH, IHD, AMI, and osteoporosis complications using regularized and weighted random survival forests model

**Fig. 3:**
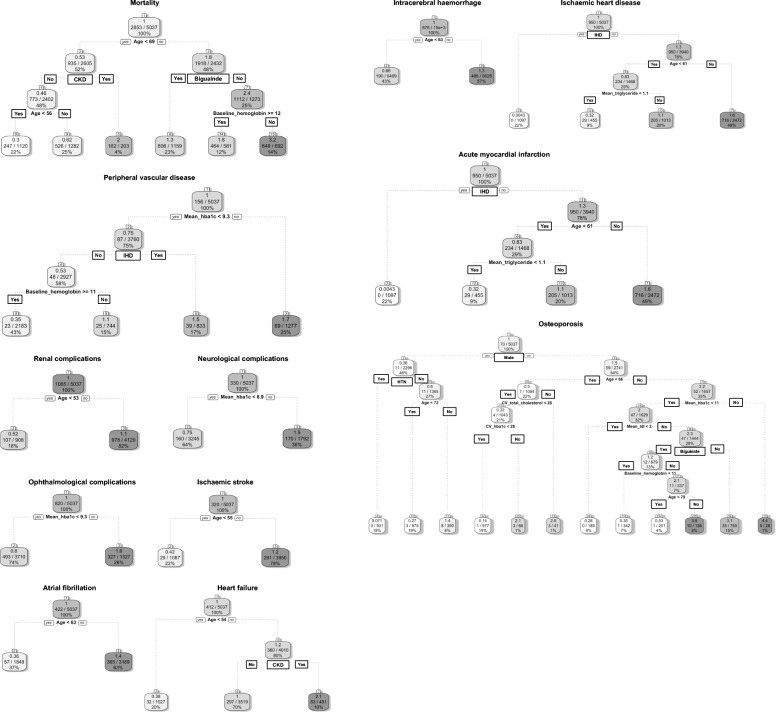
**a** Main tree based decision rules to predict mortality, renal, PVD, and neurological complications using regularized and weighted random survival forests model. **b** Main tree based decision rules to predict ophthalmological, ischemic stroke, AF, and HF complications using regularized and weighted random survival forests model. **c** Main tree based decision rules to predict ICH, IHD, AMI, and osteoporosis complications using regularized and weighted random survival forests model

A regularized and weighted RSF model was devised, with significant variables identified from univariate Cox regression inputted. This yielded the importance ranking and minimal depth of each variable in the tree structure of the model, as shown in Fig. 2 a for mortality, renal, PVD, and neurological complications, Fig. 2 b for ophthalmological, ischemic stroke, AF, and HF complications, and Fig. 2 c for ICH, IHD, AMI, and osteoporosis complications. The corresponding decision rules derived by using the regularized and weighted random survival forests model were generated based on the out-of-bag validation dataset (*N* = 5037; Fig. 3 a, b and c). The minimal depth assumes that variables with high impact on the prediction are those that most frequently split nodes nearest to the root node, where they partition the largest samples of the population. Minimal depth measures important risk factors by averaging the depth of the first split for each variable over all trees within the forest. Smaller minimal depth values indicate that the variable separates large groups of observations, and therefore has a large impact on the prediction.

The performance of the model for survival analysis of each complication outcome is compared with baselines including RSF and Cox models, based on a five-fold cross-validation approach (Table 7). According to the evaluation metric of Harrell’s C-index, our model outperforms both RSF and Cox for survival analysis of all-cause mortality, renal complications, PVD, ischemic stroke, AF, HF, ICH, IHD, AMI, and osteoporosis complications, and almost the same for dementia, neurological, ophthalmological, and complications. The model also shows higher prediction accuracy according to evaluation metrics of precision, recall, and AUC.

**Table 7:** Model performance comparison analyses with five-fold cross validation

	Our model				RSF				Cox			
	Precision	Recall	AUC	C-index	Precision	Recall	AUC	C-index	Precision	Recall	AUC	C-index
Mortality	0.9212	0.8663	0.8986	**0.8804**	0.8468	0.8962	0.8377	0.8178	0.7576	0.8025	0.7221	0.7676
Renal	0.9237	0.9180	0.8763	**0.8269**	0.8855	0.8563	0.8577	0.8194	0.7625	0.7803	0.8008	0.7470
PVD	0.8913	0.8565	0.8880	**0.8701**	0.8922	0.8617	0.8517	0.7701	0.7779	0.7289	0.7517	0.7848
Neurological	0.9104	0.9252	0.9111	0.8318	0.8842	0.8223	0.8480	**0.8511**	0.7874	0.7434	0.7969	0.7706
Ophthalmological	0.8902	0.8766	0.9065	0.8208	0.8557	0.8234	0.8643	**0.8237**	0.7671	0.7522	0.7814	0.7517
Ischemic stroke	0.8998	0.9010	0.8885	**0.8527**	0.8367	0.8983	0.8634	0.8484	0.7925	0.7752	0.7884	0.7538
AF	0.9352	0.8641	0.8998	**0.8740**	0.8194	0.8733	0.8523	0.8125	0.7747	0.7571	0.7742	0.7647
HF	0.8963	0.9175	0.8947	**0.8943**	0.8700	0.8533	0.8330	0.7767	0.8047	0.7708	0.7585	0.7749
ICH	0.7893	0.7992	0.7156	**0.7154**	0.7918	0.7796	0.7034	0.7070	0.6405	0.6590	0.6857	0.6414
IHD	0.8829	0.8948	0.9108	**0.8528**	0.8775	0.8375	0.8328	0.87964	0.7720	0.7579	0.7985	0.7738
AMI	0.9073	0.9077	0.8861	**0.8386**	0.8689	0.8455	0.8246	0.7782	0.7815	0.7640	0.7499	0.7845
Osteoporosis	0.7341	0.7142	0.7014	**0.6372**	0.6565	0.7487	0.7372	0.6244	0.6760	0.6717	0.6890	0.5857
Dementia	0.8837	0.8651	0.8660	0.8784	0.8354	0.8594	0.8549	**0.8790**	0.7566	0.7575	0.7345	0.7772

*PVD* Peripheral vascular disease, *AF* Atrial fibrillation, *HF* Heart failure, *ICH* Intracranial hemorrhage, *IHD* Ischemic heart disease, *AMI* Acute myocardial infarction

## Discussion

There are several major findings of the present study: 1) HbA1c and lipid variability can be used to evaluate the risk for a diverse range of adverse outcomes in diabetes; 2) HbA1c variability is positively associated with increased NLR and frequency of hypoglycemia episode; 3) there are interactions present between the value and variability of different lipid parameters.

Although HbA1c and lipid indices were assumed to show a positive linear correlation with mortality risk, there is emerging evidence suggesting that the mortality risk increases at the extreme ends of the parameters. Currie et al. first demonstrated the increase in cardiovascular event incidence and all-cause mortality under both low and high mean HbA1c in 2010, which explained the increased mortality under aggressive glycemic control in clinical trials [31, 32]. Subsequent cohort studies provided further evidence for the J-shaped association between mean HbA1c and all-cause mortality [33–35]. Furthermore, recent studies have found that similar to HbA1c, a U-shaped relationship is demonstrated between the lipid indices and adverse outcomes [36–38]. These findings explain the “reverse epidemiology” observed in both the present study and existing studies, where risk factors for the outcome lower the event risk instead, such as the lowering of intracranial hemorrhage and AF risk under raised mean LDL-C in this cohort [39]. Overall, the J-shaped associations justify the heterogenous predictions by mean HbA1c and lipid indices.

Heterogeneity is also demonstrated in the prediction findings of HDL-C variability. Currently, research on the predictive value of HDL-C variability is limited and yields conflicting findings. Whilst some studies report greater risk for adverse events under increased HDL-C variability, others reported insignificant findings [40–44]. Furthermore, as suggested by prior studies, the reflection of lifestyle changes by HDL-C variability may be a contributing factor, where the difference in the effect of interaction between lifestyle factors such as smoking, alcoholism, and physical activity lead to the varied predictive value of HDL-C variability across different outcomes [44, 45]. Since SD is positively correlated to the mean, given the value and variability of HDL-C yields opposite effects, the effects of variability may be reduced when SD is used as a measure of variability [40]. The standardization of variability measures can encourage the application of parameters of variability into clinical practice.

Although the mechanism behind HbA1c and lipid variability is unclear, several hypotheses were raised and explored. Large scale cohort studies have demonstrated the association between HbA1c variability with all-cause mortality and other adverse outcomes [46–48]. In terms of HbA1c variability, it is proposed that its relationship to intermittent hypoglycemia underlies the increased mortality risk. Indeed, our team recently reported a significant relationship between the frequency of hypoglycemia episodes and HbA1c variability, with the latter predicting all-cause mortality, cardiovascular-specific mortality and various diabetic-related complications [49]. Besides mortality due to hypoglycemia, a common and lethal complication in diabetes, intermittent hypoglycemia induces a higher level of oxidative stress [50, 51], causing endothelial dysfunction and chronic inflammation, ultimately leading to increased mortality risk [52–54]. It has been reported that both acute and chronic glycemic variability can induce oxidative stress and lead to chronic inflammation [55]. Indeed, increased metabolic variability can induce damage to different organs, leading to complications such as heart failure [56]. The present study provides supporting evidence for the hypothesis by demonstrating a significant association between HbA1c variability, hypoglycemic frequency, and baseline NLR. Other than NLR, further inflammatory markers such as C-reactive protein were found to be associated with HbA1c variability [57]. Similar to HbA1c, the mechanism for lipid variability to increase mortality risk is speculated to be associated with induced oxidative stress. It is speculated that large fluctuations in both LDL-C and HDL-C can lead to plaque instability, therefore releases atherogenic substances and therefore increase mortality risk [19, 58]. The significant association between baseline NLR and variability across different lipid indices provide insights towards the proposed underlying mechanisms between lipid variability and chronic inflammation. Additionally, the increased variability across biomarkers may reflect generalized frailty [19].

The effects of anti-diabetic agents on the risk of adverse events in diabetic patients have been well studied [59]. In agreement with the present study, sulphonylurea use has been reported to raise the risk of mortality, cardiovascular events, and renal impairment significantly [60–62]. It should be noted that the use of add-on therapy to insulin may indicate more severe diabetes or used to slow the progression of complications. Hence the drug-use is the effect, rather than the cause of the adverse outcome. This may explain the increased ophthalmological complication and cardiovascular event risk in biguanide and alpha-glucosidase inhibitors in the present study, contrary to the cardiovascular protective effects reported by existing studies [63–65]. Additionally, the insignificant effect of DPP4 inhibitors and thiazolidinedione may be attributed to the fewer number of patients prescribed with these drugs in the present cohort. Previously, thiazolidinediones have been associated with a greater risk of heart failure. In our study, this was associated with a lower risk of heart failure on univariate Cox regression, but not after propensity score matching for other antidiabetic drugs (unpublished results). Nevertheless, thiazolidinedione has been associated with beneficial effects such as reducing the incidence of atrial fibrillation [66], which are explicable by reverse remodeling [67–70]. Finally, the annualized mortality rate in our study was 5.87% in our cohort, compared to 3.4% in another local study [71]. The reason is that our study cohort included only diabetic patients who received insulin therapy, which would invariably include those at the highest risk. Moreover, the inclusion of patients who were already on insulin therapy in 2009 meant that few patients benefited from newer anti-diabetic drug classes such as SGLT2 inhibitors, which have been associated with lower mortality [72].

Statistical methods such as classification and regression trees are commonly used and is familiar for clinicians but are limited by high variance and poor performance [73, 74]. These can be overcome by RSF, which builds hundreds of tree branches and outputs the results by voting [28]. RSF reduces variance and bias by using all the collected variables, then automatically assess the nonlinear effects and complex interactions amongst them [22]. RSF is fully non-parametric, including the effects of the treatments and predictor variables, whereas traditional methods such as Cox model utilize a linear combination of attributes [75]. RSF has been applied in serval risk stratification models for different diseases [76–82], and has been shown to outperform classical statistical methods, such as the Cox-proportional hazards models [76, 83].

Our study demonstrates the principle that machine learning algorithms can further improve risk prediction of time-to-event (mortality and complications) in diabetic patients receiving insulin therapy. The generated importance rankings and minimal depths of prognostic risk variables can be applied in clinical practice as an easy-for-use complication score for early survival risk identification. Through complication-specific risk stratification amongst diabetic patients, a personalized management approach with close monitoring for specific complications that individual patients are high risk of can be adopted.

### Strengths and limitations

The major strengths of the present study include: 1) the effects of clinical and biochemical parameters on adverse effects were assessed using a large population-based dataset; 2) the risk for a diverse range of adverse events in diabetes is evaluated; 3) interrelations between chronic inflammation and both HbA1c and lipid variability is explored to provide insights on the underlying mechanisms in the pathogenesis; 4) variability is examined by more than one measure to limit the effects of inherent bias; 5) long follow-up period allows for the capture of serial variability and long term adverse outcome.

Several limitations should be noted for the present study. Firstly, similar to other observational studies, there is potential under-coding, missing data, and coding error. Moreover, observational studies can only establish correlation, not causation. Furthermore, the duration of diabetes was not accounted for. However, given that all patients in the study cohort were prescribed insulin for glycemic control, an advanced stage of diabetes can be inferred. Moreover, there is a large change in the management guidelines, therapeutic options, and treatment targets throughout follow-up. Additionally, there is a lack of data on the patient’s body mass index and lifestyle factors, such as smoking, alcoholism, and diet, from the database. These variables may affect the lipid levels, in particular HDL-C. The analysis of all-cause mortality is especially affected, given the wide range of contributing factors and influential effect of lifestyle choices. Finally, as the main aim of this study was to examine the predictive values of HbA1c or lipid variability for adverse outcomes, the initial analyses on the relationships between these variability indices, NLR and hypoglycemia were exploratory. The inter-relationships between these variables, including the use of mediation analysis, will be explored in future studies.

## Conclusion

In conclusion, the present study demonstrates that high HbA1c and lipid variability is associated with an increased risk for adverse outcomes in diabetes across different organ systems. The association between hypoglycemic frequency and baseline NLR with HbA1c and lipid variability suggests that intermittent hypoglycemia and chronic inflammation contribute to the mechanism underlying the pathogenic effect of fluctuating glycated hemoglobin and lipid levels. Future studies on the interactions between lipid variability can help to facilitate the application of variability measures in clinical risk stratification. The effects of the sequence of diabetic adverse outcomes on the ultimate patient survival can be explored to gain insights on the systemic pathogenesis of diabetes.

## Supplementary Information

**Additional file 1: Supplementary Table 1.** ICD-9 of Outcome and Pre-existing Comorbidities. **Supplementary Table 2.** Extracted parameters of patient data. **Supplementary Table 3.** Univariate Cox Regression for Adverse Outcomes.

## Data Availability

The data of this study are available upon reasonable request to the corresponding author.
