# Supplementary material for: Glycemic and lipid variability for predicting complications and mortality in diabetes mellitus using machine learning
Source: BMC Endocr Disord. 2021 May 4;21:94. doi: 10.1186/s12902-021-00751-4 (PMC8097996; doi:10.1186/s12902-021-00751-4)
Supplement: Supplementary file 1 — Additional file 1: Supplementary Table 1. ICD-9 of Outcome and Pre-existing Comorbidities. Supplementary Table 2. Extracted parameters of patient data. Supplementary Table 3. Univariate Cox Regression for Adverse Outcomes. [file 12902_2021_751_MOESM1_ESM.pdf]

Title Page: Prediction of Complications and All-cause Mortality with Glycemic and Lipid Variability in Diabetes Mellitus Using Machine Learning

Sharen Lee * [1], Jiandong Zhou * [2], Wing Tak Wong PhD [3], Tong Liu MD PhD [4], William KK Wu PhD FRCPath FRCP [5], Ian Chi Kei Wong PhD [6, 7], Qingpeng Zhang PhD # [2], Gary Tse PhD FRCP FFPH # [3, 7]

[1] Cardiovascular Analytics Group, Laboratory of Cardiovascular Physiology, Li Ka Shing Institute of Health Sciences, Hong Kong, China
[2] School of Data Science, City University of Hong Kong, Hong Kong, China
[3] School of Life Sciences, Chinese University of Hong Kong, Hong Kong S.A.R., China
[4] Tianjin Key Laboratory of Ionic-Molecular Function of Cardiovascular disease, Department of Cardiology, Tianjin Institute of Cardiology, Second Hospital of Tianjin Medical University, Tianjin 300211, China
[5] Li Ka Shing Institute of Health Sciences, Faculty of Medicine, The Chinese University of Hong Kong, Hong Kong, China
[6] Department of Pharmacology and Pharmacy, University of Hong Kong, Pokfulam, Hong Kong, China
Medicines Optimisation Research and Education (CMORE), UCL School of Pharmacy, London, United Kingdom
[7] Faculty of Health and Medical Sciences, University of Surrey, GU2 7AL, Guildford, United Kingdom

Co-first authors

# Correspondence to:

Dr. Qingpeng Zhang, PhD

School of Data Science, City University of Hong Kong,

Hong Kong SAR, China

Email: qingpeng.zhang@cityu.edu.hk

Dr. Gary Tse, PhD, FRCP, FFPH

Tianjin Key Laboratory of Ionic-Molecular Function of Cardiovascular disease, Department of Cardiology, Tianjin Institute of Cardiology, Second Hospital of Tianjin Medical University, Tianjin 300211, China

Faculty of Health and Medical Sciences, University of Surrey, GU2 7AL, Guildford, United Kingdom

Email: g.tse@surrey.ac.uk

Supplementary Details

Description of anti-diabetic drugs

The mean daily insulin dosage regimen of the cohort is 20.2 ± 12.6 units per day. Other classes of anti-diabetic agents prescribed include: 1) biguanide (n= 14 522, mean daily dose= 1682 ± 882 mg/day), 2) sulphonylurea (n= 10 459, mean daily dose= 191 ± 312mg/day), 3) thiazolidinedione (n= 890, mean daily dose= 7.59 ± 9.03mg/day), 4) alpha-glucosidase inhibitor (n= 751, mean daily dose= 138 ± 128mg/day), 5) DPP4 inhibitor (n= 113, mean daily dose= 91.6 ± 27.5mg), 6) GLP agonist (n= 13, mean daily dose= 11.9 ± 3.96mg/day), 7) meglitinide (n= 9, mean daily dose= 1.17 ± 0.887mg/day). The following classes of cardiovascular medications were prescribed: 1) ACEI/ ARB (n= 15 059, mean daily dose= 17.4 ± 37.0mg/day), 2) CCB (n= 10 986, mean daily dose= 60.0 ± 50.6mg/day), 3) lipid-lowering agents (n= 10 685, mean daily dose= 23.3 ± 68.4 mg/day), 4) aspirin (n= 9114, mean daily dose= 102 ± 54.1 mg/day), 5) diuretic (n= 7 349, mean daily dose= 40.3 ± 64.2mg/day), 6) beta-adrenergic receptor blocker (n= 7 082, mean daily dose= 79.4 ± 81.6mg/day).

Description of machine learning results

There may be slight difference between importance ranking and minimal depth sequence, although they both can be used to identify the highly predictive risk variables. Both importance ranking and minimal depth were used to uncover the most important variables for predicting time-to-event complication outcomes.

The results showed that age, CKD, baseline hemoglobin, biguanide, and AMI are the five most predictive risk variables to predict all-cause mortality. Moreover, age is the most important variable for renal complication prediction, followed by baseline hemoglobin, HF, AMI, and IHD. AMI, CKD, age, stroke and mean Hba1c are much predictive for PVD prediction. Age, biguanide, IHD, baseline hemoglobin shows great importance for neurological complication. IHD, biguanide, standard deviation of HbA1c, and mean triglyceride are the most five predictive risk factors to identify

ophthalmological complication. For predicting ischemic stroke, age, HF, HT, stroke, IHD are significantly predictive. Age, CKD, AMI, IHD, HF, and HT shows much informative for AF outcome prediction. While CKD, AMI, age, baseline hemoglobin and mean HDL were very useful to predict HF outcome. For ICH prediction, patient’s age, baseline hemoglobin, CKD, HT, mean IDL are more predictive, while age, AMI, HF, baseline hemoglobin, and mean triglyceride are the most important for IHD outcome prediction. AMI, HF, age, CKD, and baseline hemoglobin are the most informative for AMI outcome prediction, while patient’s age, gender, hypoglycemia, and mean HDL is useful for osteoporosis prediction.

Supplementary Table 1. ICD-9 of Outcome and Pre-existing Comorbidities

	ICD Code
Type 1 Diabetes Mellitus	250.01
Outcome	
Neurological Complications	250.6
Ophthalmological Complications	250.5
Renal Complications	250.4
Peripheral Vascular Disease	250.7
Ischemic Stroke and Transient Ischemic Attack	433.01, 433.11, 433.21, 433.31, 433.81, 433.91, 434.01, 434.91, 436, 435
Intracranial Hemorrhage	430.00, 431.00, 432.00, 432.10, 432.90, 852.00-852.09, 852.20-852.29, 852.40-852.49, 853.00
Atrial Fibrillation	427.31, 429.4
Heart Failure	428.00-428.99
Ischemic Heart Disease	410.00-411.99, 413.00-414.99
Acute Myocardial Infarction	410.00-410.99
Osteoporosis	733.00
Dementia	290.00-290.99, 331.00, 294.10-294.29, 331.19
Pre-existing Comorbidity	
Chronic Renal Disease	585.00-585.99
Chronic Obstructive Pulmonary Disease	490.00-496.99
Heart Failure	398.91, 402.91, 428.00-428.99
Ischemic Heart Disease	411.00-411.99, 413.00-414.99
Hypertension	401.9
Chronic Liver Disease	570.00-573.99
Acute Myocardial Infarction	410.00-410.99, 412.00-412.99
Stroke	430.00-438.99

Supplementary Table 2. Extracted parameters of patient data

Category	Parameters
Demographic	Age<br>Male<br>Type 1 Diabetes Mellitus
Comorbidities	Hypertension<br>Ophthalmological Complications<br>Ischemic Heart Disease<br>Ischemic Stroke and Transient Ischemic Attack<br>Heart Failure<br>Chronic Renal Disease<br>Chronic Liver Disease<br>Acute Myocardial Infarction<br>Chronic Obstructive Pulmonary Disease
Anti-diabetic Medication	Biguanide<br>Sulphonylurea<br>Thiazolidinedione<br>Alpha-Glucosidase<br>Dipeptidyl Peptidase-4 Inhibitor<br>Glucagon-Like Peptide-1 Receptor Agonist
Cardiovascular Medication	Angiotensinogen-Converting Enzyme Inhibitor/ Angiotensin-Receptor Blocker<br>Calcium Channel Blocker<br>Lipid-Lowering Agents<br>Aspirin<br>Diuretic<br>Beta-Adrenergic Receptor Blocker
Urinalysis	Albumin/Creatinine Ratio (mg/mmol)<br>Creatinine Clearance (ml/min)<br>Spot Protein (g/d)<br>Spot Albumin (mg/L)<br>Spot Glucose (mmol/L)<br>24-hours Total Protein (g/d)<br>24-hours Total Albumin (mg/d)
Glycemic and Lipid Profile	Fasting Glucose (mmol/L)<br>Random Glucose (mmol/L)<br>HbA1c (%)<br>Total Cholesterol (mmol/L)<br>High Density Lipoprotein (HDL) Cholesterol (mmol/L)<br>Calculated Low Density Lipoprotein (LDL) Cholesterol (mmol/L)<br>Direct LDL Cholesterol (mmol/L)<br>Triglyceride (mmol/L)
Renal and Liver Function Test	Creatinine (umol/L)<br>Sodium (mmol/L)<br>Potassium (mmol/L)<br>Urate (umol/L)<br>Urea (mmol/L)<br>Albumin (g/L)<br>Alanine Aminotransferase (ALT) (U/L)<br>Alkaline Phosphatase (ALP) (U/L)<br>Total Bilirubin (umol/L)<br>Total Protein (g/L)
Complete Blood Count	Hemoglobin (g/dL)

Mean Corpuscular Hemoglobin (MCH) (pg)
Mean Corpuscular Hemoglobin Concentration (MCHC) (g/dL)
Mean Corpuscular Volume (MCV) (fL)
Hematocrit (L/L)
Basophil (x109/L)
Eosinophil (x109/L)
Lymphocyte (x109/L)
Monocyte (x109/L)
Neutrophil (x109/L)
Platelet (x109/L)
Red Blood Cell (x1012/L)
White Blood Cell (x109/L)

Supplementary Table 3. Univariate Cox Regression for Adverse Outcomes

Predictor	Hazard Ratio (HR)	95% Confidence Interval (CI)	P-Value
Neurological Complications			
Age	1.01	[1.00, 1.01]	**<0.001**
Male	1.47	[1.34, 1.62]	**<0.0001**
Diabetes Duration	1.04	[1.03, 1.06]	**<0.0001**
HbA1c			
Baseline	0.999	[0.974, 1.02]	0.934
Mean	1.18	[1.14, 1.22]	**<0.0001**
Standard Deviation	1.13	[1.07, 1.19]	**<0.0001**
Coefficient of Variation	1.01	[1.00, 1.01]	**0.014**
Total Cholesterol			
Baseline	0.960	[0.917, 1.01]	0.081
Mean	1.06	[1.00, 1.12]	**0.042**
Standard Deviation	1.16	[1.06, 1.27]	**0.001**
Coefficient of Variation	1.01	[1.00, 1.02]	**0.001**
HDL Cholesterol			
Baseline	1.01	[0.891, 1.15]	0.863
Mean	0.634	[0.544, 0.741]	**<0.0001**
Standard Deviation	0.555	[0.323, 0.954]	**0.033**
Coefficient of Variation	1.00	[0.994, 1.01]	0.881
LDL Cholesterol			
Baseline	1.00	[0.946, 1.06]	0.964
Mean	1.03	[0.961, 1.11]	0.398
Standard Deviation	1.27	[1.11, 1.45]	**<0.001**
Coefficient of Variation	1.01	[1.00, 1.01]	**0.001**
Triglyceride			
Baseline	0.962	[0.928, 0.997]	**0.035**
Mean	1.07	[1.05, 1.10]	**<0.0001**
Standard Deviation	1.05	[1.02, 1.09]	**0.002**
Coefficient of Variation	1.00	[0.999, 1.00]	0.229
Baseline NLR	0.994	[0.981, 1.01]	0.357
Baseline Hemoglobin Count	0.945	[0.923, 0.968]	**<0.0001**
Baseline Anemia	1.17	[1.06, 1.29]	**0.002**
Hypoglycemia Frequency	1.01	[0.977, 1.04]	0.612
Anti-Diabetic Agent			
Sulphonylurea	0.936	[0.853, 1.03]	0.162
Biguanide	0.978	[0.892, 1.07]	0.629
DPP4 Inhibitor	0.923	[0.392, 1.73]	0.608
Thiazolidinedione	0.718	[0.539, 0.956]	**0.023**
Alpha-Glucosidase Inhibitor	1.05	[0.812, 1.37]	0.694
Ophthalmological Complications			
Age	0.999	[0.997, 1.00]	0.427
Male	1.01	[0.955, 1.07]	0.694
Diabetes Duration	1.11	[1.10, 1.12]	**<0.0001**
HbA1c			
Baseline	1.01	[0.997, 1.03]	0.109

Mean	1.25	[1.23, 1.28]	**<0.0001**
Standard Deviation	1.07	[1.04, 1.11]	**<0.0001**
Coefficient of Variation	0.999	[0.995, 1.00]	0.508
Total Cholesterol			
Baseline	0.996	[0.969, 1.03]	0.804
Mean	1.10	[1.06, 1.14]	**<0.0001**
Standard Deviation	1.06	[0.994, 1.13]	0.077
Coefficient of Variation	1.00	[0.997, 1.01]	0.760
HDL Cholesterol			
Baseline	0.993	[0.917, 1.08]	0.870
Mean	0.842	[0.768, 0.923]	**<0.001**
Standard Deviation	0.432	[0.305, 0.613]	**<0.0001**
Coefficient of Variation	0.990	[0.986, 0.995]	**<0.0001**
LDL Cholesterol			
Baseline	0.994	[0.959, 1.03]	0.720
Mean	1.05	[1.00, 1.10]	**0.032**
Standard Deviation	1.06	[0.974, 1.16]	0.170
Coefficient of Variation	1.00	[0.999, 1.00]	0.342
Triglyceride			
Baseline	1.01	[0.993, 1.03]	0.245
Mean	1.07	[1.05, 1.08]	**<0.0001**
Standard Deviation	1.05	[1.03, 1.07]	**<0.0001**
Coefficient of Variation	0.999	[0.997, 1.00]	0.287
Baseline NLR	0.986	[0.977, 0.995]	**0.003**
Baseline Hemoglobin Count	1.01	[0.993, 1.03]	0.265
Baseline Anemia	0.939	[0.881, 1.00]	0.051
Hypoglycemia Frequency	0.949	[0.926, 0.972]	**<0.0001**
Anti-Diabetic Agent			
Sulphonylurea	0.972	[0.918, 1.03]	0.344
Biguanide	1.41	[1.33, 1.50]	**<0.0001**
DPP4 Inhibitor	1.01	[0.660, 1.56]	0.954
Thiazolidinedione	1.09	[0.937, 1.26]	0.271
Alpha-Glucosidase Inhibitor	0.948	[0.798, 1.13]	0.540
Renal Complications			
Age	1.02	[1.02, 1.02]	**<0.0001**
Male	1.12	[1.06, 1.18]	**<0.0001**
Diabetes Duration	1.05	[1.04, 1.06]	**<0.0001**
HbA1c			
Baseline	1.00	[0.987, 1.02]	0.813
Mean	1.13	[1.11, 1.15]	**<0.0001**
Standard Deviation	1.07	[1.04, 1.10]	**<0.0001**
Coefficient of Variation	1.00	[1.00, 1.01]	0.089
Total Cholesterol			
Baseline	0.999	[0.973, 1.03]	0.943
Mean	1.08	[1.05, 1.12]	**<0.0001**
Standard Deviation	1.18	[1.11, 1.24]	**<0.0001**
Coefficient of Variation	1.01	[1.01, 1.01]	**<0.0001**

HDL Cholesterol			
Baseline	1.00	[0.929, 1.08]	0.979
Mean	0.510	[0.464, 0.560]	**<0.0001**
Standard Deviation	0.372	[0.268, 0.516]	**<0.0001**
Coefficient of Variation	1.00	[0.996, 1.00]	0.906
LDL Cholesterol			
Baseline	1.00	[0.969, 1.04]	0.942
Mean	1.04	[0.998, 1.09]	0.062
Standard Deviation	1.27	[1.18, 1.38]	**<0.0001**
Coefficient of Variation	1.01	[1.01, 1.01]	**<0.0001**
Triglyceride			
Baseline	0.989	[0.970, 1.01]	0.231
Mean	1.10	[1.08, 1.11]	**<0.0001**
Standard Deviation	1.06	[1.04, 1.08]	**<0.0001**
Coefficient of Variation	1.00	[1.00, 1.00]	**0.045**
Baseline NLR	1.00	[0.994, 1.01]	0.821
Baseline Hemoglobin Count	0.908	[0.896, 0.921]	**<0.0001**
Baseline Anemia	1.46	[1.38, 1.55]	**<0.0001**
Hypoglycemia Frequency	1.00	[0.982, 1.02]	0.865
Anti-Diabetic Agent			
Sulphonylurea	1.29	[1.22, 1.36]	**<0.0001**
Biguanide	0.970	[0.920, 1.02]	0.275
DPP4 Inhibitor	0.761	[0.485, 1.19]	0.234
Thiazolidinedione	1.00	[0.867, 1.16]	0.986
Alpha-Glucosidase Inhibitor	1.14	[0.986, 1.33]	0.075
Dementia			
Age	1.08	[1.07, 1.08]	**<0.0001**
Male	0.717	[0.630, 0.815]	**<0.0001**
Diabetes Duration	0.981	[0.956, 1.01]	0.140
HbA1c			
Baseline	1.00	[0.968, 1.04]	0.891
Mean	1.00	[0.953, 1.05]	0.963
Standard Deviation	1.11	[1.03, 1.19]	**0.005**
Coefficient of Variation	1.01	[1.00, 1.02]	**0.003**
Total Cholesterol			
Baseline	0.984	[0.922, 1.05]	0.617
Mean	0.945	[0.871, 1.03]	0.181
Standard Deviation	1.01	[0.864, 1.17]	0.935
Coefficient of Variation	1.00	[0.995, 1.01]	0.411
HDL Cholesterol			
Baseline	1.02	[0.852, 1.23]	0.818
Mean	0.956	[0.779, 1.17]	0.664
Standard Deviation	1.35	[0.660, 2.74]	0.414
Coefficient of Variation	1.01	[0.995, 1.02]	0.342
LDL Cholesterol			
Baseline	1.05	[0.971, 1.14]	0.220
Mean	0.925	[0.835, 1.03]	0.140
Standard Deviation	1.17	[0.960, 1.42]	0.119

Coefficient of Variation	1.01	[1.00, 1.01]	**0.013**
Triglyceride			
Baseline	0.990	[0.946, 1.04]	0.643
Mean	1.00	[0.956, 1.05]	0.877
Standard Deviation	0.981	[0.917, 1.05]	0.569
Coefficient of Variation	0.999	[0.995, 1.00]	0.532
Baseline NLR	1.01	[0.993, 1.02]	0.302
Baseline Hemoglobin Count	0.927	[0.897, 0.957]	**<0.0001**
Baseline Anemia	1.43	[1.25, 1.64]	**<0.0001**
Hypoglycemia Frequency	1.01	[0.964, 1.05]	0.723
Anti-Diabetic Agent			
Sulphonylurea	1.22	[1.08, 1.39]	**0.002**
Biguanide	0.962	[0.846, 1.09]	0.548
DPP4 Inhibitor	0.459	[0.115, 1.84]	0.271
Thiazolidinedione	0.857	[0.592, 1.24]	0.414
Alpha-Glucosidase Inhibitor	0.729	[0.473, 1.12]	0.152
Osteoporosis			
Age	1.06	[1.04, 1.07]	**<0.0001**
Male	0.249	[0.186, 0.335]	**<0.0001**
Diabetes Duration	1.01	[0.961, 1.06]	0.776
HbA1c			
Baseline	0.957	[0.893, 1.03]	0.205
Mean	1.00	[0.916, 1.10]	0.943
Standard Deviation	0.879	[0.747, 1.04]	0.123
Coefficient of Variation	0.985	[0.969, 1.00]	0.059
Total Cholesterol			
Baseline	0.997	[0.885, 1.12]	0.954
Mean	1.06	[0.918, 1.23]	0.411
Standard Deviation	0.966	[0.720, 1.30]	0.819
Coefficient of Variation	0.997	[0.981, 1.01]	0.757
HDL Cholesterol			
Baseline	1.01	[0.723, 1.41]	0.958
Mean	1.78	[1.29, 2.44]	**<0.001**
Standard Deviation	1.49	[0.412, 5.42]	0.542
Coefficient of Variation	0.987	[0.968, 1.01]	0.222
LDL Cholesterol			
Baseline	1.04	[0.902, 1.21]	0.566
Mean	1.03	[0.854, 1.24]	0.773
Standard Deviation	0.940	[0.639, 1.38]	0.752
Coefficient of Variation	0.996	[0.985, 1.01]	0.489
Triglyceride			
Baseline	1.04	[0.991, 1.09]	0.117
Mean	0.918	[0.818, 1.03]	0.145
Standard Deviation	0.855	[0.708, 1.03]	0.105
Coefficient of Variation	0.990	[0.981, 0.998]	**0.020**
Baseline NLR	0.986	[0.951, 1.02]	0.454
Baseline Hemoglobin Count	0.853	[0.803, 0.906]	**<0.0001**

Baseline Anemia	1.71	[1.32, 2.21]	**<0.0001**
Hypoglycemia Frequency	1.04	[0.969, 1.12]	0.263
Anti-Diabetic Agent			
Sulphonylurea	0.924	[0.726, 1.18]	0.522
Biguanide	1.21	[0.948, 1.54]	0.127
DPP4 Inhibitor	0.808	[0.113, 5.76]	0.831
Thiazolidinedione	0.608	[0.271, 1.37]	0.228
Alpha-Glucosidase Inhibitor	1.10	[0.567, 2.14]	0.775
Peripheral Vascular Disease			
Age	1.02	[1.01, 1.02]	**<0.0001**
Male	1.36	[1.17, 1.58]	**<0.0001**
Diabetes Duration	0.968	[0.939, 0.998]	**0.035**
HbA1c			
Baseline	1.03	[0.987, 1.07]	0.195
Mean	1.20	[1.14, 1.26]	**<0.0001**
Standard Deviation	1.19	[1.11, 1.28]	**<0.0001**
Coefficient of Variation	1.01	[1.01, 1.02]	**0.001**
Total Cholesterol			
Baseline	0.938	[0.870, 1.01]	0.095
Mean	1.14	[1.05, 1.25]	**0.003**
Standard Deviation	1.34	[1.21, 1.50]	**<0.0001**
Coefficient of Variation	1.02	[1.01, 1.03]	**<0.0001**
HDL Cholesterol			
Baseline	1.10	[0.905, 1.35]	0.330
Mean	0.452	[0.346, 0.590]	**<0.0001**
Standard Deviation	1.82	[0.840, 3.93]	0.130
Coefficient of Variation	1.03	[1.02, 1.03]	**<0.0001**
LDL Cholesterol			
Baseline	0.937	[0.853, 1.03]	0.177
Mean	1.11	[0.994, 1.24]	0.065
Standard Deviation	1.54	[1.26, 1.88]	**<0.0001**
Coefficient of Variation	1.01	[1.01, 1.02]	**<0.001**
Triglyceride			
Baseline	1.00	[0.959, 1.05]	0.898
Mean	1.09	[1.06, 1.13]	**<0.0001**
Standard Deviation	1.09	[1.05, 1.13]	**<0.0001**
Coefficient of Variation	1.01	[1.00, 1.01]	**<0.0001**
Baseline NLR	1.02	[1.00, 1.03]	**0.016**
Baseline Hemoglobin Count	0.868	[0.836, 0.901]	**<0.0001**
Baseline Anemia	1.71	[1.46, 2.01]	**<0.0001**
Hypoglycemia Frequency	1.07	[1.03, 1.12]	**0.001**
Anti-Diabetic Agent			
Sulphonylurea	0.897	[0.772, 1.04]	0.159
Biguanide	0.768	[0.663, 0.889]	0.889
DPP4 Inhibitor	0.943	[0.304, 2.93]	0.919
Thiazolidinedione	0.952	[0.634, 1.43]	0.812
Alpha-Glucosidase Inhibitor	1.81	[1.30, 2.52]	**<0.001**

Intracranial Hemorrhage			
Age	1.03	[1.02, 1.03]	**<0.0001**
Male	1.20	[1.06, 1.35]	**0.004**
Diabetes Duration	0.966	[0.942, 0.990]	**0.006**
HbA1c			
Baseline	1.02	[0.988, 1.05]	0.218
Mean	0.991	[0.945, 1.04]	0.703
Standard Deviation	1.04	[0.970, 1.12]	0.265
Coefficient of Variation	1.01	[0.998, 1.01]	0.168
Total Cholesterol			
Baseline	0.950	[0.893, 1.01]	0.104
Mean	0.945	[0.875, 1.02]	0.155
Standard Deviation	1.13	[1.00, 1.29]	**0.050**
Coefficient of Variation	1.01	[1.00, 1.02]	**0.026**
HDL Cholesterol			
Baseline	0.980	[0.826, 1.16]	0.815
Mean	0.757	[0.619, 0.926]	**0.007**
Standard Deviation	1.17	[0.598, 2.30]	0.644
Coefficient of Variation	1.01	[1.00, 1.02]	**0.020**
LDL Cholesterol			
Baseline	0.975	[0.902, 1.05]	0.513
Mean	0.875	[0.794, 0.964]	**0.007**
Standard Deviation	1.15	[0.954, 1.38]	0.145
Coefficient of Variation	1.01	[1.00, 1.01]	**0.008**
Triglyceride			
Baseline	0.994	[0.955, 1.04]	0.776
Mean	1.07	[1.03, 1.10]	**<0.001**
Standard Deviation	1.05	[1.00, 1.10]	**0.033**
Coefficient of Variation	1.00	[0.998, 1.01]	0.462
Baseline NLR	1.00	[0.989, 1.02]	0.623
Baseline Hemoglobin Count	0.923	[0.894, 0.952]	**<0.0001**
Baseline Anemia	1.19	[1.05, 1.35]	**0.008**
Hypoglycemia Frequency	1.04	[0.997, 1.08]	0.075
Anti-Diabetic Agent			
Sulphonylurea	1.17	[1.04, 1.32]	**0.012**
Biguanide	0.875	[0.775, 0.988]	**0.031**
DPP4 Inhibitor	0.850	[0.318, 2.27]	0.746
Thiazolidinedione	1.05	[0.763, 1.45]	0.764
Alpha-Glucosidase Inhibitor	0.887	[0.610, 1.29]	0.533
Ischemic Stroke and Transient Ischemic Attack/ Transient Ischemic Attack			
Age	1.03	[1.03, 1.04]	**<0.0001**
Male	0.998	[0.897, 1.11]	0.964
Diabetes Duration	0.989	[0.968, 1.01]	0.327
HbA1c			
Baseline	0.984	[0.954, 1.01]	0.291
Mean	1.05	[1.01, 1.09]	**0.024**
Standard Deviation	0.994	[0.929, 1.06]	0.854
Coefficient of Variation	0.997	[0.990, 1.00]	0.350

Total Cholesterol			
Baseline	0.987	[0.935, 1.04]	0.637
Mean	1.03	[0.967, 1.11]	0.329
Standard Deviation	1.10	[0.977, 1.24]	0.114
Coefficient of Variation	1.01	[0.999, 1.01]	0.113
HDL Cholesterol			
Baseline	1.01	[0.872, 1.18]	0.868
Mean	0.800	[0.671, 0.954]	**0.013**
Standard Deviation	1.20	[0.660, 2.18]	0.551
Coefficient of Variation	1.01	[1.00, 1.02]	0.061
LDL Cholesterol			
Baseline	0.967	[0.903, 1.04]	0.335
Mean	1.00	[0.922, 1.09]	0.942
Standard Deviation	1.14	[0.962, 1.34]	0.133
Coefficient of Variation	1.00	[0.999, 1.01]	0.119
Triglyceride			
Baseline	0.981	[0.943, 1.02]	0.342
Mean	1.05	[1.02, 1.09]	**0.003**
Standard Deviation	1.03	[0.985, 1.08]	0.193
Coefficient of Variation	1.00	[0.997, 1.00]	0.995
Baseline NLR	1.01	[0.992, 1.02]	0.404
Baseline Hemoglobin Count	0.986	[0.958, 1.02]	0.337
Baseline Anemia	1.07	[0.956, 1.20]	0.232
Hypoglycemia Frequency	1.00	[0.963, 1.04]	0.963
Anti-Diabetic Agent			
Sulphonylurea	1.19	[1.07, 1.33]	**0.001**
Biguanide	1.18	[1.06, 1.32]	**0.003**
DPP4 Inhibitor	0.491	[0.158, 1.52]	0.218
Thiazolidinedione	0.765	[0.551, 1.06]	0.107
Alpha-Glucosidase Inhibitor	0.891	[0.640, 1.24]	0.496
Ischemic Heart Disease			
Age	1.02	[1.02, 1.02]	**<0.0001**
Male	1.03	[0.975, 1.10]	0.266
Diabetes Duration	1.02	[1.01, 1.04]	**<0.0001**
HbA1c			
Baseline	0.996	[0.980, 1.01]	0.647
Mean	1.05	[1.03, 1.08]	**<0.0001**
Standard Deviation	0.987	[0.951, 1.02]	0.470
Coefficient of Variation	0.996	[0.993, 1.00]	**0.046**
Total Cholesterol			
Baseline	1.01	[0.978, 1.04]	0.634
Mean	1.25	[1.21, 1.30]	**<0.0001**
Standard Deviation	1.22	[1.16, 1.29]	**<0.0001**
Coefficient of Variation	1.01	[1.01, 1.01]	**<0.0001**
HDL Cholesterol			
Baseline	1.12	[1.03, 1.21]	**0.006**
Mean	0.642	[0.581, 0.710]	**<0.0001**

Standard Deviation	0.596	[0.421, 0.844]	**0.004**
Coefficient of Variation	1.00	[0.998, 1.01]	0.366
LDL Cholesterol			
Baseline	1.01	[0.972, 1.05]	0.675
Mean	1.27	[1.22, 1.33]	**<0.0001**
Standard Deviation	1.43	[1.32, 1.56]	**<0.0001**
Coefficient of Variation	1.01	[1.00, 1.01]	**<0.0001**
Triglyceride			
Baseline	0.981	[0.960, 1.00]	0.084
Mean	1.09	[1.07, 1.10]	**<0.0001**
Standard Deviation	1.05	[1.02, 1.07]	**<0.0001**
Coefficient of Variation	1.00	[0.999, 1.00]	0.313
Baseline NLR	1.00	[0.996, 1.01]	0.379
Baseline Hemoglobin Count	0.925	[0.911, 0.939]	**<0.0001**
Baseline Anemia	1.29	[1.21, 1.38]	**<0.0001**
Hypoglycemia Frequency	1.00	[0.979, 1.02]	0.983
Anti-Diabetic Agent			
Sulphonylurea	1.16	[1.09, 1.23]	**<0.0001**
Biguanide	1.10	[1.03, 1.16]	**0.003**
DPP4 Inhibitor	1.13	[0.752, 1.71]	0.553
Thiazolidinedione	1.13	[0.974, 1.31]	0.107
Alpha-Glucosidase Inhibitor	1.20	[1.03, 1.41]	**0.023**
Acute Myocardial Infarction			
Age	1.03	[1.03, 1.03]	**<0.0001**
Male	0.80	[0.914, 1.05]	0.562
Diabetes Duration	0.989	[0.975, 1.00]	0.103
HbA1c			
Baseline	1.01	[0.986, 1.03]	0.603
Mean	1.07	[1.04, 1.10]	**<0.0001**
Standard Deviation	1.01	[0.966, 1.05]	0.726
Coefficient of Variation	0.998	[0.994, 1.00]	0.338
Total Cholesterol			
Baseline	0.990	[0.956, 1.03]	0.562
Mean	1.18	[1.13, 1.23]	**<0.0001**
Standard Deviation	1.22	[1.14, 1.30]	**<0.0001**
Coefficient of Variation	1.01	[1.01, 1.02]	**<0.0001**
HDL Cholesterol			
Baseline	0.992	[0.900, 1.09]	0.867
Mean	0.522	[0.462, 0.591]	**<0.0001**
Standard Deviation	0.680	[0.453, 1.02]	0.064
Coefficient of Variation	1.01	[1.00, 1.01]	**0.005**
LDL Cholesterol			
Baseline	0.982	[0.940, 1.03]	0.430
Mean	1.18	[1.12, 1.24]	**<0.0001**
Standard Deviation	1.41	[1.28, 1.56]	**<0.0001**
Coefficient of Variation	1.01	[1.00, 1.01]	**<0.0001**
Triglyceride			

Baseline	0.977	[0.952, 1.00]	0.076
Mean	1.09	[1.08, 1.11]	**<0.0001**
Standard Deviation	1.06	[1.03, 1.08]	**<0.0001**
Coefficient of Variation	1.00	[1.00, 1.00]	**0.042**
Baseline NLR	1.00	[0.993, 1.01]	0.601
Baseline Hemoglobin Count	0.895	[0.879, 0.911]	**<0.0001**
Baseline Anemia	1.48	[1.38, 1.60]	**<0.0001**
Hypoglycemia Frequency	1.01	[0.982, 1.03]	0.619
Anti-Diabetic Agent			
Sulphonylurea	1.22	[1.14, 1.31]	**<0.0001**
Biguanide	0.961	[0.896, 1.03]	0.271
DPP4 Inhibitor	0.836	[0.474, 1.47]	0.535
Thiazolidinedione	0.868	[0.710, 1.06]	0.164
Alpha-Glucosidase Inhibitor	1.23	[1.02, 1.48]	**0.032**
Atrial Fibrillation			
Age	1.04	[1.04, 1.05]	**<0.0001**
Male	0.884	[0.807, 0.968]	**0.008**
Diabetes Duration	0.989	[0.975, 1.00]	0.103
HbA1c			
Baseline	1.00	[0.975, 1.03]	0.975
Mean	0.961	[0.926, 0.996]	**0.029**
Standard Deviation	1.05	[0.993, 1.11]	0.090
Coefficient of Variation	1.01	[1.00, 1.01]	**0.024**
Total Cholesterol			
Baseline	1.03	[0.990, 1.08]	0.134
Mean	0.889	[0.838, 0.943]	**<0.0001**
Standard Deviation	1.12	[1.02, 1.23]	**0.020**
Coefficient of Variation	1.01	[1.01, 1.02]	**<0.001**
HDL Cholesterol			
Baseline	1.02	[0.894, 1.15]	0.823
Mean	0.667	[0.572, 0.779]	**<0.0001**
Standard Deviation	0.988	[0.590, 1.65]	0.962
Coefficient of Variation	1.01	[1.00, 1.02]	**0.005**
LDL Cholesterol			
Baseline	1.03	[0.969, 1.09]	0.389
Mean	0.832	[0.773, 0.895]	**<0.0001**
Standard Deviation	1.20	[1.04, 1.37]	**0.011**
Coefficient of Variation	1.01	[1.01, 1.01]	**<0.0001**
Triglyceride			
Baseline	0.982	[0.950, 1.01]	0.266
Mean	1.04	[1.01, 1.07]	**0.019**
Standard Deviation	0.998	[0.955, 1.04]	0.914
Coefficient of Variation	0.998	[0.995, 1.00]	0.147
Baseline NLR	1.00	[0.992, 1.02]	0.574
Baseline Hemoglobin Count	0.907	[0.886, 0.929]	**<0.0001**
Baseline Anemia	1.41	[1.28, 1.56]	**<0.0001**
Hypoglycemia Frequency	1.03	[1.00, 1.06]	**0.030**

Anti-Diabetic Agent			
Sulphonylurea	1.17	[1.07, 1.28]	**0.001**
Biguanide	0.933	[0.851, 1.02]	0.136
DPP4 Inhibitor	0.716	[0.321, 1.60]	0.413
Thiazolidinedione	0.965	[0.751, 1.24]	0.781
Alpha-Glucosidase Inhibitor	1.00	[0.765, 1.31]	0.997
Heart Failure			
Age	1.03	[1.03, 1.04]	**<0.0001**
Male	1.27	[1.17, 1.39]	**<0.0001**
Diabetes Duration	0.937	[0.919, 0.955]	**<0.0001**
HbA1c			
Baseline	1.02	[0.993, 1.04]	0.170
Mean	1.05	[1.02, 1.09]	**0.004**
Standard Deviation	1.10	[1.04, 1.16]	**<0.001**
Coefficient of Variation	1.01	[1.00, 1.01]	**0.001**
Total Cholesterol			
Baseline	0.999	[0.954, 1.05]	0.961
Mean	1.06	[0.997, 1.12]	0.065
Standard Deviation	1.28	[1.19, 1.38]	**<0.0001**
Coefficient of Variation	1.02	[1.01, 1.03]	**<0.0001**
HDL Cholesterol			
Baseline	0.976	[0.857, 1.11]	0.709
Mean	0.417	[0.352, 0.494]	**<0.0001**
Standard Deviation	1.25	[0.754, 2.07]	0.387
Coefficient of Variation	1.02	[1.02, 1.03]	**<0.0001**
LDL Cholesterol			
Baseline	1.02	[0.962, 1.08]	0.517
Mean	1.06	[0.988, 1.14]	0.103
Standard Deviation	1.51	[1.33, 1.71]	**<0.0001**
Coefficient of Variation	1.01	[1.01, 1.02]	**<0.0001**
Triglyceride			
Baseline	1.00	[0.973, 1.03]	0.930
Mean	1.09	[1.07, 1.12]	**<0.0001**
Standard Deviation	1.06	[1.03, 1.09]	**<0.001**
Coefficient of Variation	1.00	[1.00, 1.01]	**0.006**
Baseline NLR	1.02	[1.01, 1.03]	**<0.0001**
Baseline Hemoglobin Count	0.835	[0.815, 0.854]	**<0.0001**
Baseline Anemia	1.85	[1.67, 2.04]	**<0.0001**
Hypoglycemia Frequency	1.08	[1.06, 1.11]	**<0.0001**
Anti-Diabetic Agent			
Sulphonylurea	1.30	[1.19, 1.43]	**<0.0001**
Biguanide	0.618	[0.563, 0.678]	**<0.0001**
DPP4 Inhibitor	0.608	[0.253, 1.46]	0.266
Thiazolidinedione	0.722	[0.540, 0.964]	**0.027**
Alpha-Glucosidase Inhibitor	1.17	[0.905, 1.50]	0.235
All-Cause Mortality			
Age	1.06	[1.06, 1.06]	**<0.0001**

Male	1.14	[1.10, 1.18]	**<0.0001**
Diabetes Duration	0.930	[0.923, 0.936]	**<0.0001**
HbA1c			
Baseline	0.997	[0.988, 1.01]	0.549
Mean	0.964	[0.951, 0.977]	**<0.0001**
Standard Deviation	1.11	[1.09, 1.14]	**<0.0001**
Coefficient of Variation	1.01	[1.01, 1.01]	**<0.0001**
Total Cholesterol			
Baseline	0.992	[0.976, 1.01]	0.340
Mean	0.965	[0.943, 0.988]	**0.003**
Standard Deviation	1.29	[1.25, 1.33]	**<0.0001**
Coefficient of Variation	1.02	[1.02, 1.03]	**<0.0001**
HDL Cholesterol			
Baseline	0.964	[0.921, 1.01]	0.114
Mean	0.571	[0.537, 0.607]	**<0.0001**
Standard Deviation	3.11	[2.60, 3.72]	**<0.0001**
Coefficient of Variation	0.970	[0.927, 1.02]	0.194
LDL Cholesterol			
Baseline	1.02	[0.995, 1.04]	0.131
Mean	0.919	[0.893, 0.946]	**<0.0001**
Standard Deviation	1.56	[1.48, 1.64]	**<0.0001**
Coefficient of Variation	1.02	[1.01, 1.02]	**<0.0001**
Triglyceride			
Baseline	0.989	[0.978, 1.00]	0.0557
Mean	1.06	[1.05, 1.07]	**<0.0001**
Standard Deviation	1.03	[1.02, 1.04]	**<0.0001**
Coefficient of Variation	1.00	[1.00, 1.00]	**0.002**
Baseline NLR	1.03	[1.03, 1.04]	**<0.0001**
Baseline Hemoglobin Count	0.794	[0.786, 0.801]	**<0.0001**
Baseline Anemia	2.11	[2.04, 2.20]	**<0.0001**
Hypoglycemia Frequency	1.08	[1.06, 1.09]	**<0.0001**
Anti-Diabetic Agent			
Sulphonylurea	1.13	[1.09, 1.17]	**<0.0001**
Biguanide	0.541	[0.522, 0.560]	**<0.0001**
DPP4 Inhibitor	0.450	[0.324, 0.655]	**<0.0001**
Thiazolidinedione	0.703	[0.632, 0.782]	**<0.0001**
Alpha-Glucosidase Inhibitor	1.05	[0.953, 1.17]	0.305
